# Oral Health of Women and Children: Progress, Challenges, and Priorities

**DOI:** 10.1007/s10995-023-03757-7

**Published:** 2023-07-21

**Authors:** Jayanth Kumar, James J. Crall, Katrina Holt

**Affiliations:** 1 Department of Public Health, MS 7208, 1616 Capitol Avenue, Sacramento, CA 95814 USA; 2 UCLA School of Dentistry, Los Angeles, CA 90095 USA; 3 Department of Pediatrics, National Maternal and Child Oral Health Resource Center, Georgetown University, 37th and O Streets, N.W, Washington, DC 20057 USA

**Keywords:** Oral diseases, Maternal and Child Health, Title V, Community water fluoridation, School dental program, Medicaid

## Abstract

**Introduction:**

Tooth decay remains the most prevalent chronic disease in children and adults, even though it is largely preventable. Studies show that mothers’ oral and overall health is linked to children’s oral health and pregnancy outcomes. This paper examines achievements during the last 20 years, assesses current challenges, and discusses future priorities.

**Oral health status:**

Data show a modest improvement in children’s oral health during the last 20 years; however, tooth decay still affects more than half of adolescents. According to national survey data, about 26% of working-age adults had untreated tooth decay. Overall, significant oral health disparities by race/ethnicity and income persist.

**Dental service utilization:**

The annual dental visit rate for children in the 2015 Medical Expenditure Panel Survey was 48%. Among children enrolled in Medicaid, dental visit rates increased from 18% in 1993 to nearly 50% in 2018. About 46% of women are estimated to receive teeth cleaning during pregnancy. Over the years, race or ethnicity and income-level differences in dental visits observed in the early 2000s have narrowed substantially in children but not among pregnant women.

**Discussion:**

Many effective interventions are available at the community and individual levels but are underutilized. Lack of integration of oral health into the overall health care system and programs, community conditions, poverty, and limited health literacy make it difficult for families to engage in healthy habits, use preventive interventions, and access treatment promptly.

**Conclusion:**

To further improve oral health, policy and system reforms are needed to address the factors mentioned above. Therefore, we urge the federal Maternal and Child Health Bureau to take steps to convene a workshop to develop a framework for future actions.

## Significance

*What is already known on this subject*? Existing publications discuss the extent to which women, children, and adolescents experience oral health problems, effective policies and programs, and the need to integrate oral health care into the overall health care system.

*What does this study add*? This manuscript provides a historical perspective on efforts and progress made to address the causes of high oral disease rates, unmet oral health treatment needs, and profound oral health disparities. It also discusses challenges that four major programs have encountered in working to achieve better oral health outcomes. In addition, it makes recommendations for improving oral health and calls for a national workshop to develop a framework for future actions.

## Introduction

During World War II, one of the primary reasons for rejection of army recruits was failure to meet the minimum standard of having at least 12 teeth (Allen, 1992). Today, few young people have missing permanent teeth. Nevertheless, despite dramatic declines in tooth decay prevalence and severity in school-aged children during the 1970 and 1980 s, tooth decay remains the most prevalent chronic disease affecting children, and despite some documented reductions, oral health disparities remain a cause for concern (National Institutes of Health, 2021).

As early as the 1920s, the Maternal and Child Health (MCH) Section of the American Public Health Association (APHA) recognized the importance of oral health to overall health by including “dental hygiene” in its efforts to detect and correct health defects in children (Schlesinger & Stitt, 1974). Warren and Kavanagh have thoroughly documented the role the federal Maternal and Child Health Bureau (MCHB) has played over 110 years in improving the health and well-being of America’s mothers, children, and families (Warren & Kavanagh, 2023). While there is much to celebrate regarding the improved oral health in the United States over these years, much more needs to be done to promote oral health and provide early, ongoing and integrated oral health care to women during and after pregnancy and to children beginning within the first year of life.

This paper examines achievements made during the past 20 years in maternal and child oral health and oral health care, assesses current challenges, and discusses future priorities for achieving more effective, efficient, and uniform oral health improvements.

### Oral Health Status of Children

Among children aged 5 to 17, the mean number of decayed, missing, and filled permanent tooth surfaces (DMFS) decreased by about 56%, from 7.1 in 1971–1974 to 3.1 in 1986–1987 (Brown et al., 1994; Brunelle & Carlos, 1990). Approximately 50% of children were reported to have caries-free permanent teeth in 1987, and those with decay had fewer decayed surfaces on average (Brunelle & Carlos, 1990). The widespread adoption of community water fluoridation, use of fluoride toothpaste, promotion of daily toothbrushing habits, and improvements in clinical prevention and treatment services beginning in the mid-1960s led to substantial reductions in tooth decay in children in the latter half of the last century.

More recently, the 2021 report *Oral Health in America: Advances and Challenges* found only a modest improvement in the overall oral health of children and adolescents during the past 20 years (National Institutes of Health, 2021). Tooth decay remains a prevalent chronic disease affecting about 2 in 10 children aged 2–5 years, 5 in 10 children aged 6–11 years, and more than half of adolescents (Centers for Disease Control and Prevention, 2019; Dye et al., 2017). That report also noted that tooth decay disproportionately affects population subgroups with limited economic resources and low educational attainment. For example, the prevalence of tooth decay in permanent teeth among all adolescents aged 12–19 years was 56.8% but was 65% among children from families with low incomes. The prevalence was also higher among Mexican American children (68.9%) than among non-Hispanic children (54.3% white, non-Hispanic; 57.1% black, non-Hispanic) (National Institutes of Health, 2021).

The most significant improvement in oral health status during the past 20 years is the substantial decline in untreated tooth decay among children aged 2–5 years, especially among those from disadvantaged groups (National Institutes of Health, 2021). Overall, nearly 10% of children have untreated tooth decay now compared to 20% of children about two decades ago (Centers for Disease Control and Prevention, 2019). However, similar reductions in the prevalence of tooth decay and in untreated tooth decay among American Indian and Alaska Native children were not observed during the past decade (Phipps & Ricks, 2014).

Among adolescents aged 12–19 years, the prevalence of untreated tooth decay decreased from 20% during 1999–2004 to 17% during 2011–2016, with the most significant decline (8% points) among Mexican American adolescents (Centers for Disease Control and Prevention, 2019). However, the prevalence of untreated tooth decay was twice as high among adolescents from families considered poor and near-poor combined (22%) compared to adolescents from higher-income families (11%).

Slade and Sanders reported that oral health disparities related to household income worsened for children and adolescents in three age groups across three time points from 1988 to 2014 (Slade & Sanders, 2018). For example, during 1988 − 1994, children aged 12–17 years from families living below the poverty threshold had 1.3 more DMFS than their counterparts from families with income at least three times the poverty threshold. By 2011–2014, the disparity had increased to 2.9 DMFS. Moreover, it is unclear whether improvements in younger cohorts will lead to future reductions in disparities in untreated tooth decay among adolescents.

### Oral Health Status of Women

During the past 20 years, increasing attention has been paid to improving the oral health of pregnant women and mothers because of the dual impact on women’s health and children’s health. Health education and promotion activities are crucial for improving feeding practices, decreasing oral infection to reduce the transmission of caries-causing bacteria from caregivers to their children, and preventing the spread of infection and inflammatory byproducts to the fetus. Pregnancy and the perinatal period also are increasingly recognized as periods of elevated risk for oral diseases in women (National Institutes of Health, 2021). Because data from national surveys about the prevalence of dental diseases among women of childbearing age are limited, this paper discusses the oral health status of all women.

The national prevalence of (untreated and treated) tooth decay, untreated tooth decay, and mean DMFT among women with teeth aged 20–64 years, as reported by the 2011–2016 National Health and Nutrition Examination Survey (NHANES), were 92%, 24%, and 9.6, respectively. There were no noticeable gender-specific differences (Centers for Disease Control and Prevention, 2019).

The 2011–2016 NHANES data showed significant disparities by race/ethnicity and income. Untreated tooth decay was observed in 22% of white, non-Hispanic adults; 37% of Mexican-American adults; and 40% of black, non-Hispanic adults. Among adults with a household income at least twice the federal poverty level, 18% had untreated tooth decay compared with 41% of adults with lower household incomes (Centers for Disease Control and Prevention, 2019). Most women of childbearing age had close to the full complement of teeth.

Azofeifa et al., analyzed 1999–2004 NHANES data on tooth decay and periodontal disease among pregnant and non-pregnant women of reproductive age (Azofeifa et al., 2016). The prevalence of periodontal disease among Mexican-American pregnant and non-pregnant women (9.3% and 7.6%) was higher than the corresponding prevalence (3.1% and 4.2%) for all pregnant and non-pregnant women. The authors did not observe statistically significant differences in the prevalence of tooth decay or periodontal disease between pregnant and non-pregnant women but concluded that women with less education or lower family income had a higher prevalence of untreated tooth decay, severe tooth decay, and periodontal disease compared to women with more education or higher family income.

### Dental Visits and Utilization of Preventive Oral Health Services

The annual dental visit rate for children in the 2015 Medical Expenditure Panel Survey (MEPS) was 48% (Manski et al., 2022). Among children enrolled in Medicaid, dental services increased from 18% to 1993 to nearly 50% in 2018 (Crall & Vujicic, 2020). Similar increases in dental visit rates also were observed in the National Health Interview Survey (National Center for Health Statistics, 2021). Analyses conducted by the American Dental Association Health Policy Institute found that in 2016 a higher percentage (67.1%) of children enrolled in private insurance visited a dentist in the past year, compared to 50.4% of children enrolled in Medicaid or the Children’s Health Insurance Program (CHIP) (American Dental Association, Health Policy Institute, 2018). Over the years, race/ethnicity and income-level differences seen in the early 2000s have narrowed substantially.

Dental sealants provide an effective way to prevent tooth decay on the chewing surfaces of molar (back) teeth. According to the 2011–2016 NHANES, nearly half of children aged 9–11 years (51%) and adolescents aged 12–19 years (48%) had dental sealants on permanent teeth, reflecting an increase of more than 10% points since 1999–2004 (Centers for Disease Control and Prevention, 2019). In addition, increases of 12% points or more were found among all disadvantaged groups, indicating that disparities in the prevalence of dental sealants have narrowed somewhat.

According to Pregnancy Risk Assessment Monitoring System (PRAMS) 2019 self-reported dental visit data, about 46% of respondents reported having their teeth cleaned during pregnancy (Centers for Disease Control and Prevention, 2020a). Analysis of PRAMS 2012–2015 dental visit data from 31 states and New York City showed that 51.7% of women reported a dental visit for teeth cleaning during their most recent pregnancy, an improvement from the PRAMS 2004-06 findings (Lee et al., 2022). However, in California and North Carolina, dental visit rates in pregnant women did not improve over the last decade (Kumar & Samelson, 2022; Stephens et al., 2020; Walton-Haynes et al., 2022). The dental visit rate among states varied from a low of 35.2% in Georgia to a high of 65.6% in Massachusetts, with a substantial gap in dental visits for teeth cleaning between women enrolled in Medicaid (38.3%) and those enrolled in private insurance (61.4%). There were also racial and ethnic disparities in dental visit rates and unmet oral health needs, with Black, non-Hispanic women (59.5%) and Hispanic women (63.7%) less likely to have their oral health needs met compared with White, non-Hispanic women (67.7%). Disparities in utilization of oral health services during pregnancy by age, race/ethnicity, geographic region, income, and educational levels have been observed in several states (Moss et al., 2021; Naavaal et al., 2019; Naavaal & Harless, 2022; Stephens et al., 2020; Walton-Haynes et al., 2022).

### Dental Insurance or Benefit Coverage

Dental insurance or benefits can be a significant factor in increasing access to and utilization of oral health care that reduces unmet oral health needs. Analysis of MEPS data showed that approximately 88% of children from birth to age 20 had dental benefit coverage in 2015, increasing from 73% in 1996 (Manski et al., 2022). Data from the Centers for Medicare & Medicaid Services (CMS) indicate that 38,593,210 children aged 20 and younger were enrolled in Medicaid for at least 90 continuous days in FY 2020 (Centers for Medicare and Medicaid Services, 2019).

According to 2021 National Health Interview Survey self-reported data, approximately 67.8% of U.S. adults aged 18–44 years had dental insurance coverage (Office of Disease Prevention and Health Promotion, 2022). According to PRAMS 2020 self-reported data, the percentage of women with health insurance coverage during pregnancy was 97%, with nearly 37% having public health insurance (Centers for Disease Control and Prevention, 2020a). However, not all health insurance plans include dental benefits; and the extent of dental benefits can vary significantly. More recently, CMS has reported that as of October 2022, all state Medicaid programs provide some dental benefits for pregnant and postpartum women (Centers for Medicare & Medicaid Services, 2022).

### Other Conditions

Cleft lip and cleft palate are common birth defects, often accompanied by malocclusion and nasal deformities. The Centers for Disease Control and Prevention (CDC) estimates that each year about 1 in 1,564 babies are born with cleft lip and cleft palate; another 1 in 2,807 babies are born with a cleft lip; and 1 in 1,687 are born with a cleft palate (Centers for Disease Control and Prevention, 2022a).

Human papillomavirus (HPV) is the most common sexually transmitted infection in the United States. According to the CDC, each year about 14,800 new cases of oropharynx cancer are caused by human papillomavirus (HPV) (Centers for Disease Control and Prevention, 2022b). The 2021 *Oral Health in America: Advances and Challenges* recommended that oral health professionals who see adolescent patients should counsel their parents about the HPV vaccine and HPV’s link to oropharyngeal cancer.

## A Historical Perspective

The National Maternal and Child Oral Health Resource Center developed the *Leadership and Legacy: Oral Health Milestones in Maternal and Child Health* (*Leadership and Legacy*) timeline that provides a historical perspective of significant events in the United States (National Maternal and Child Oral Health Resource Center, 2012–). The timeline (Table 1) was adapted to provide an overview of efforts to address the causes of high oral disease rates, unmet treatment needs, and profound oral health disparities. The most significant event of the American Public Health Association MCH Section is the formation of the Committee on Public Health Dentistry in 1936 and the subsequent report on the scope of dental health activities and the organizational units (Schlesinger & Stitt, 1974). According to Schlesinger and Stitt, the report suggested various activities in the dental health program that included oral health education and care for children, women during pregnancy and lactation, and adults to relieve pain and eradicate infection.

**Table 1:** Oral health milestones in maternal and child oral health

1840	First dental school established
1880	Importance of dental hygiene gains acceptance
1906	First free school dental clinic opened
1912	Children’s Bureau established
1912	Maternal and Child Health Research Program established
1921	American Public Health Association established Child Hygiene Section (now known as the Maternal and Child Health Section)
1921	Maternity and Infancy Care Act (commonly known as the Sheppard-Towner Act) enacted
1931	Fluoride found to reduce dental caries
1935	Title V of the Social Security Act enacted
1936	APHA Child Hygiene Section established Committee on Public Health Dentistry
1937	American Association of Public Health Dentistry established
1943	American Public Health Association, Oral Health Section, established
1945	Fluoridation of community water studies began
1948	Association of State and Territorial Dental Directors established
1955	Indian Health Service established
1962	National Institute of Child Health and Human Development established
1964	Head Start established, initially as an 8-week summer program
1965	Maternal and Infant Care Projects established
1965	Medicaid (Title XIX) established
1965	Neighborhood Health Centers Projects (commonly known as community health centers) established
1967	Medicaid amended to create the Early, Periodic, Diagnostic, and Treatment (EPSDT) program
1967	Title V established three clinical grant programs: dental care, family planning, and neonatal intensive care
1967	Dental sealants developed
1972	National Health Service Corps created
1979	*Healthy People: The Surgeon General’s Report on Health Promotion and Disease Prevention (report) released*
1980	*Public Policy Options for Better Dental Health: Report of a Study released*
1982	The First International Conference on the Declining Prevalence of Dental Caries held
1983	*Consensus Development Conference Statement on Dental Sealants in the Prevention of Tooth Decay (report) published*
1989	Public Health Service Workshop on Oral Health of Mothers and Children held
1990	Children’s Health Insurance Program (CHIP) established
1993	Government Performance and Results Act passed
1994	Medical providers begin delivering preventive oral health services
1996	*Children’s Dental Services Under Medicaid: Access and Utilization (report) released*
1996	Health Centers Consolidation Act enacted
1996	National Maternal and Child Oral Health Resource Center established
1997	State Children’s Health Insurance Program established
1998	Building Partnerships to Improve Children’s Access to Medicaid Oral Health Services (conference) held
1998	Head Start Program Performance Standard to promote oral hygiene (toothbrushing) established
1998	Health Resources and Services Administration / Health Care Financing Administration Oral Health intra-agency agreement established
2000	The Face of a Child: Surgeon General’s Conference on Children and Oral Health held
2000	*Oral Health in America: A Report of the Surgeon General released*
2000	Partnership for State Oral Health Leadership Program established
2001	Head Start Bureau / Maternal and Child Health Bureau (MCHB) agreement established
2003	*A National Call to Action to Promote Oral Health: A Public-Private Partnership Under the Leadership of the Office of the Surgeon General (report) released*
2003	National Maternal and Child Oral Health Policy Center established
2008	MCHB funded the Targeted Oral Health Service Systems (TOHSS) grant program
2010	The Patient Protection and Affordable Care Act (ACA) requires health insurance exchanges (health insurance markets) to offer oral health coverage for children
2011	*Advancing Oral Health in America (report) released*
2011	*Improving Access and Utilization of Oral Health Services for Children in Medicaid and CHIP Programs: CMS Oral Health Strategy (report) released*
2011	*Improving Access to Oral Health Care for Vulnerable and Underserved Populations (report) released*
2011	School-Based Comprehensive Oral Health Services Program established
2012	*Oral Health Care During Pregnancy: A National Consensus Statement—Summary of an Expert Workgroup Meeting released*
2013	*Oral Health Literacy—Workshop Summary released*
2013	Perinatal and Infant Oral Health Quality Improvement Initiative established
2015	Title V MCH Services Block Grant transformation
2017	Center for Oral Health Systems Integration and Improvement Consortium established
2019	Networks for Oral Health Integration Within the Maternal and Child Health Safety Net Program established
2021	*Oral Health in America: Advances and Challenges (report) released*

Source: Adapted from *Leadership and Legacy: Oral Health Milestones in Maternal and Child Oral Health (mchoralhealth.org)*

The Public Health Service Workshop on Oral Health of Mothers and Children held in 1989 is another landmark event that outlined the need for local, state, and national initiatives to promote, coordinate, and integrate effective oral health activities for children and mothers (National Center for Education in Maternal and Child Health, 1989; Steffensen, 1990). It also emphasized the need to identify and promote coordinated strategies to maximize resources for prevention, education, services, and research to ensure continued progress in oral health. The workshop identified several factors that influenced oral health outcomes: oral health policy, integration of oral health, standards, resources, documentation, evaluation, and research. Efforts to address these factors have led to the development of a system to deliver essential public health services, including effective and equitable delivery of oral health preventive and treatment services to the maternal and child population. In addition, there are factors at the individual, family, and community level that affect oral health outcomes, such as (1) knowledge, attitude, and behavior; (2) socio-geographic factors; (3) environmental and physical barriers; (4) access and availability of services, transportation, and childcare; and (5) failure to consider the acceptability of services from consumers’ perspective. Fisher-Owens et al. developed a conceptual model that identified genetic and biological factors, social and physical environments, health behaviors, and dental and medical care (Fisher-Owens et al., 2007).

The 2000 *Oral Health in America: A Report of the Surgeon General* proposed a framework for a national oral health plan to improve quality of life and eliminate oral health disparities by facilitating collaborations among individuals, health professionals, providers, and policymakers at all levels of society and by taking advantage of existing initiatives (U.S. Department of Health and Human Services, 2000). The components of the plan included changing perceptions regarding oral health and disease to become an accepted component of general health, accelerating evidence-based knowledge and applying science to improve oral health, building an effective health infrastructure to meet the oral health needs of all Americans, and integrating oral health effectively into overall health. To address the separation of oral health from the health care system, participants of a Surgeon General’s conference outlined steps to build constituencies for supporting broad policy changes and redirect resources to oral health (Mouradian, 2001).

The *Leadership and Legacy* timeline highlights the support for oral health components included in federal agency activities such as infrastructure and capacity, service, research, and professional development. The Association of State and Territorial Dental Directors has also developed guidelines for state dental programs to perform core public health functions (Association of State and Territorial Dental Directors, 2021). Systems and methods for surveillance and evaluation have also been developed for effective program planning at the state level. In addition, the Head Start program and the Medicaid Early Periodic Screening, Diagnosis, and Treatment program have developed standards. The following paragraphs describe four major programs and the implementation challenges facing them.

### Title V Grant Program

In many states, the Title V Maternal and Child Health Services Block Grant Program is a critical source of support for enhancing the availability and quality of services to improve oral health for pregnant women, children, and adolescents and to achieve the Healthy People national objectives. These services have included conducting needs assessments, promoting a child’s dental visit by age 1 in a primary care setting, expanding dental sealants and fluoride varnish applications, and improving Medicaid dental coverage and access to services.

Over the past two decades, MCHB has supported initiatives to improve oral health including the Targeted MCH Oral Health Service System, the School-Based Comprehensive Oral Health Services, the Perinatal and Infant Oral Health Quality Improvement (PIOHQI), the Partnership for Integrating Oral Health Care into Primary Care, and the Networks for Oral Health Integration Within the Maternal and Child Health Safety Net.

Advances toward improving oral health care for the MCH population have reduced untreated tooth decay by nearly 50% in young children and resulted in unprecedented dental coverage for pregnant women by all state Medicaid programs (National Maternal and Child Oral Health Resource Center, 2023). The Title V program has one national outcome measure (NOM) (NOM 14: Percent of children and adolescents, ages 1 through 17, who have decayed teeth or cavities in the past year.) and one national performance measure (NPM) with two parts: NPM 13.1: Percent of women who had a preventive dental visit during pregnancy and 13.2: Percent of children and adolescents, ages 1 through 17, who had a preventive dental visit in the past year.

However, the Health Resources and Services Administration (HRSA) has proposed changes to the Title V Maternal and Child Health Services Block Grant to States Program guidance for the next 5-year (2025–2030) period. One proposed change is to transition the oral health NPM to a state performance measure (SPM).

Oral health has been an NPM since 1997. If oral health is transitioned to an SPM, it will no longer be viewed as a national priority, and efforts to promote oral health care at the federal, state, and local levels will suffer. Therefore, the oral health NPM needs to be retained to highlight the profound disparities in unmet needs and stimulate oral-health-related activities at the state and local levels (National Maternal and Child Oral Health Resource Center, 2023).

### School Dental Programs

Since the early 1900s, school dental programs have played a vital role in addressing the oral health needs of children. Following the National Preventive Dentistry Demonstration Program (NPDDP) report (Klein et al., 1985), Schlossman, Brown, and Sedlack published a history of school dental programs in 1986, *The Public School in American Dentistry* to inform the policy debate (Schlossman et al., 1986). During this century, school dental programs had primarily taken five approaches: (1) classroom oral health education programs; (2) establishment of clinical preventive services provided by dental hygienists; (3) establishment of school dental clinics to provide essential treatment services by dentists; (4) promotion of self-applied fluoride mouth rinse program once a week in the late 1970s; and later on, (5) school dental sealant programs in the late 1980s. While the Great Depression decimated school social services, including school medical services, governments at all levels supported essential dental treatment services in schools to care for underserved children. According to Schlossman, Brown, and Sedlack, the support for school dental programs began to wane in the 1960s as nearly half of the school-aged children visited dentists (Schlossman et al., 1986). This improvement in dental visit rates led to the design of oral health education programs and their promotion as a primary role of school dental programs. By the 1970s, the expansion of the dental workforce to meet the needs of Medicaid beneficiaries and the establishment of neighborhood health centers likely led to a rapid decline in the number of school dental treatment programs. The NPDDP, which began around 1975 to test the cost and effectiveness of preventive dental programs, found that many school-based preventive interventions such as classroom education, topical fluoride application, and fluoride tablet and rinse programs were not cost-effective in preventing a substantial amount of tooth decay (Klein et al., 1985). Only community water fluoridation and dental sealant applications were effective in preventing caries; however, the cost of providing dental sealants to all children was more than the cost of preventing cavities in a few children. Furthermore, the NPDDP report found that schools were not as efficient a location for the delivery of preventive procedures as once thought. Because sealants were novel ideas for preventing caries on the most susceptible tooth surfaces and they were not covered benefits in dental insurance benefit programs, the promotion of school dental sealant programs as a long-lasting clinical intervention became the primary function of state dental programs.

Although the Community Preventive Services Taskforce has recommended school-based dental programs as an effective intervention to prevent dental caries, they are in a small percentage of schools with high need nationwide (ASTDD, 2022). In addition, both financial resources and logistical challenges have presented implementation problems. In addition to the lack of a sustainable funding mechanism, these include school recruitment and support, student participation, physical barriers, scheduling conflicts, workforce capacity, and limitations imposed by state regulations (AlEissa & Catania, 2022). To overcome these challenges, many states have promoted alternative solutions such as school dental screening laws and school-linked programs to connect children to a source of dental care. However, these programs are only successful if there is a system to track referral closures. California is implementing an electronic referral management system in schools, and its successful implementation could be a model for other states.

### Community Water Fluoridation

Community water fluoridation (CWF) is the controlled adjustment of the natural fluoride (F) concentration in community water supplies in the U.S. to the concentration of 0.7 mg/L F to prevent tooth decay. (U.S. Department of Health and Human Services Federal Panel on Community Water Fluoridation., 2015). In 1942, David Ast proposed a plan to test the fluoride-caries hypothesis generated through observational studies by adding sodium fluoride to drinking water and tracking the outcomes (Ast, 1943). He stated, “If this hypothesis can be determined affirmatively, it will indeed revolutionize our thinking and our approach to the solution of the dental caries problem. With conclusive positive evidence, it may be possible to effect mass protection and not have to depend on the individual to do anything about it…. The possibilities of such findings fairly stagger the imagination when the extent of dental caries today, the economic problem involved in an attempt to correct accumulated defects, and the difficulties encountered in getting persons to dentists for treatment are considered.” Since its widespread adoption in the 1970s, the percentage of the U.S. population receiving fluoridated water had increased to 63.4% by 2018 (or 73% on public water supplies) (Centers for Disease Control and Prevention, 2020b). The *Timeline for Community Water Fluoridation* documents the historical developments of CWF (Centers for Disease Control and Prevention, 2021). Efforts to add fluoride to drinking water supplies require policy, system, and environmental changes, such as passing laws, regulations, and resolutions, creating infrastructure and training, and making structural changes to the water treatment facility. Communities may encounter many challenges to implement and maintain a consistent level of fluoride in drinking water. These include knowledge of balancing the benefits and potential risks of fluoridation, political will, financial resources for initial start-up and operation costs, technical challenges faced by water treatment facilities, and community acceptance of governmental programs (Stocks et al., 2022). In 1999, the CDC named community water fluoridation as 1 of 10 great public health achievements of the 20th century because of its contribution to the dramatic decline in tooth decay over the past 75 years (Centers for Disease Control and Prevention, 1999).

### Medicaid and CHIP

Medicaid covers dental services for all child enrollees aged 20 and under as part of a comprehensive set of benefits, referred to as the Early and Periodic Screening, Diagnostic and Treatment (EPSDT) benefit. Increasing dental coverage and use of dental services by children enrolled in Medicaid and CHIP has been a major strategy for improving the oral health of children over the past 25 years, especially those at highest risk for oral diseases. Dental services for children must minimally include relief of pain and infections, restoration of teeth and maintenance of dental health. A referral to a dentist by his or her primary care provider is also required for every child in accordance with the periodicity schedule set by each state and at other intervals as medically necessary. The design and administration of Medicaid and CHIP dental benefits are highly dependent on programs organized by state agencies.

Dental care for adult Medicaid beneficiaries is an optional benefit for state Medicaid programs, with coverage ranging from no benefits in a few states to relatively extensive coverage in many states. With respect to the focus of this paper, most states offer more comprehensive dental benefits to the pregnant population than the general adult population and provide coverage to higher household income levels (Center for Medicaid CHIP Services & Division of Quality and Health Outcomes, 2023).

In 2011, the CMS identified several barriers to using dental services (Department of Health and Human Services, 2011). These included limited availability of dental providers, low reimbursement rates, administrative burdens for providers, lack of clear information for beneficiaries about dental benefits, missed dental appointments, transportation, cultural and language competency of providers, and the need for consumer education about the benefits of dental care. CMS worked with states to overcome these barriers and developed a national oral health strategy (Department of Health and Human Services, 2020).

Growth of these programs has substantially increased public dental benefits coverage for children to the point that in 2017 Medicaid and CHIP covered 39% of American children younger than age 19, with coverage by state ranging between 25% in Wyoming and 56% in New Mexico (Brooks et al., 2019). Expanded coverage and efforts to improve the performance of state Medicaid programs have contributed to a substantial positive impact nationwide on Medicaid-enrolled children’s use of dental services, increasing from 18% in fiscal year 1993 to nearly 50% in FY 2018 (Center for Medicaid CHIP Services & Division of Quality and Health Outcomes, 2023; Crall and Vujicic, 2020). Increased utilization also has been associated with a reduction in untreated tooth decay, particularly in younger lower-income children, between the 1999 -— 2004 and 2011–2016 national surveys. However, disparities in dental care use by insurance type remain (Center for Medicaid CHIP Services & Division of Quality and Health Outcomes, 2023).

## Recommendations for the Future

The 2021 *Oral Health in America: Advances and Challenges* concluded that research increasingly shows that poor oral health during pregnancy is linked to adverse health outcomes for mothers and babies (National Institutes of Health, 2021). This report also noted that – as research sheds light on the effects of early life experiences and social and environmental determinants of health – leading experts are focusing on developing innovative prevention strategies and more integrated, continuous approaches to health care, including activities that promote oral health throughout pregnancy, postpartum and the first three years of life (National Institutes of Health, 2021).

Transforming existing programs and care delivery systems to achieve more optimal outcomes will require a multidimensional approach focusing on communities, patients, providers, and policymakers. Recommended actions include conducting educational campaigns to increase public awareness and knowledge of the importance of dental care during pregnancy, improving health professionals’ education and training regarding the importance and safety of providing oral health care to pregnant women, systems improvements to foster better integration of oral health services across diverse types of health care providers and settings, and assuring dental benefits coverage throughout these critical periods (National Institutes of Health, 2021).

The groundwork outlining what can and should be done to promote oral health during pregnancy, postpartum and early childhood has already been established. In 2011, HRSA issued a landmark publication, *Oral Health Care During Pregnancy: A National Consensus Statement*, to highlight recommended services and required policy and system-level changes (Oral Health Care During Pregnancy Expert Workgroup, 2012). Subsequently, from 2013 to 2019, HRSA funded 16 states to participate in the PIOHQI initiative to implement evidence-based models of care that integrate preventive oral health care into primary care for pregnant women and infants. Lessons learned from this initiative about improving the oral health of pregnant women have been published (Holt & Barzel, 2022). Some of the lessons learned included: (1) securing a commitment from leadership is important to achieving project goals and cultivating and sustaining leadership’s interest and engagement; (2) making training modules available online (vs. offering trainings in person only) is necessary to meet staff training needs; (3) processes and procedures must be adapted locally to enable functionality at individual sites—a one-size-fits-all approach does not work; (4) policy change comes about slowly, and it is important to be ready when an opportunity for change presents itself; and (5) having oral health champions with relevant data to make the case for the importance of perinatal oral health is essential to effect change. APHA also has issued a policy statement for improving access to oral health care for pregnant women through education, integration of health services, insurance coverage, an appropriate dental workforce, and research (Table 2) (Oral Health Section, 2020).

**Table 2:** Selected challenges to improving the oral health of pregnant and postpartum women, and proposed solutions

Challenges	Proposed Solutions
Most pregnant and postpartum women are not aware of the importance of oral health care during pregnancy, behaviors that can help prevent oral health problems, and Medicaid dental benefits. Cultural differences between health professionals and pregnant and postpartum women can also present barriers to receiving oral health care.	Require accreditation bodies for primary care, dental, and allied dental education programs to include curriculum content on perinatal and infant oral health, including information about national and state guidelines.
	Encourage professional organizations to promote *Smiles for Life: A National Oral Health Curriculum* for primary care educational programs and practicing primary care professionals.
	Include an oral health program performance measure for pregnant and postpartum women as part of benchmark domains for demonstrating measurable improvement in HRSA’s Maternal and Infant Early Childhood Home Visiting Program.
	Support federal public health agencies and dental, allied dental, and primary care professional associations disseminating culturally tailored information about perinatal oral health to improve the public’s health literacy and to help the public make informed health care decisions.
Oral health education is not well integrated into community-based programs.	Advocate for community-based programs (e.g., Early Head Start, home visiting, WIC) that serve pregnant and postpartum women to provide oral health supplies; provide culturally tailored education on the importance of perinatal and infant oral health; make referrals to oral health professionals; provide care coordination; and collect, document, and report oral health findings.
Medicaid dental benefits for working-age adults, including pregnant and postpartum women, are optional and vary from state to state. Comprehensive dental benefits in state Medicaid programs are not available. Most pregnant and postpartum women do not know what Medicaid dental benefits are covered in their state.	Advocate for inclusion of comprehensive dental coverage in proposals aimed at expanding coverage or achieving universal coverage for pregnant and postpartum women.
	Advocate for comprehensive oral health care as a mandatory component of state Medicaid pregnancy- and postpartum-related benefits.
	Urge state Medicaid programs to include comprehensive dental coverage for women during pregnancy through 1 year postpartum.
	Recommend that state Medicaid programs reimburse oral health services delivered by prenatal care professionals.
	Recommend that state Medicaid dental reimbursement rates be increased to encourage more oral health professionals to participate in the program and provide oral health care to pregnant and postpartum women.
National public health surveillance systems lack robust and reliable mechanisms for collecting data on the oral health status of pregnant and postpartum women and on access to oral health care during pregnancy and postpartum.	Encourage federal public health agencies to partner to develop reliable and consistent mechanisms for collecting and sharing data on pregnant and postpartum women’s oral health status, dental insurance coverage, and access to and utilization of oral health care.
	Increase funding for public health programs and research focused on improving perinatal oral health.
There is no national oral health objective.	Include an objective on the oral health of pregnant and postpartum women in Healthy People national objectives.
There is insufficient state-level data to assess oral health status, dental insurance coverage, and access to oral health care among women during pregnancy and the postpartum period.	Encourage state and local public health agencies to partner to develop reliable and consistent mechanisms for collecting and sharing data on pregnant and postpartum women’s oral health status, dental insurance coverage, and access to oral health care.

Source: American Public Health Association. (2020). Improving access to dental care for pregnant women through education, integration of health services, insurance coverage, an appropriate dental workforce, and research. https://www.apha.org/Policies-and-Advocacy/Public-Health-Policy-Statements/Policy-Database/2021/01/12/Improving-Access-to-Dental-Care-for-Pregnant-Women.

The information in this source is the basis of the proposed solutions in this table

Crall and Vujicic have identified achievements and ongoing challenges in improving children’s oral health and offered a range of corresponding solutions (Table 3 (Crall & Vujicic, 2020). These include redesigning health care delivery systems and benefits based on chronic care models, supporting the development of model programs that emphasize early intervention and integrated care, revitalizing oral health education programs, and aligning federal and state efforts to measure and improve program performance.

**Table 3:** Selected challenges to improving children’s oral health, and proposed solutions

Challenges	Proposed solutions
The public, health professionals, and policymakers do not understand that tooth decay must be addressed using evidence-based chronic disease approaches.	Develop evidence-based approaches for increasing oral health literacy, focusing on infants and children at risk for tooth decay and their caregivers.
Professional education on oral health and oral health care delivery systems are “siloed,” and there is a lack of integrated approaches to tooth decay prevention and management.	Provide federal and state support for innovative professional education and care delivery in settings that embrace dental-medical integration and clinical excellence.
Approaches to providing oral health benefits are “siloed,” and there is a lack of support for integration of dental and oral health benefits as part of benefits for whole-person care.	Support integration of dental and oral health benefits as part of whole-person care.
There is a paucity of meaningful evidence and effective models for how to address oral health disparities for children.	Prioritize meaningful clinical and translational research, demonstration programs, and centers of excellence to address oral health disparities in children.
Data systems for monitoring disease status and the impact of clinical and population health interventions or quality-improvement efforts are inadequate.	Incentivize the use of data-collection tools that support surveillance, program evaluation, and quality improvement, including diagnostic codes to assess outcomes.
Few standardized and tested quality and performance measures have been developed or are being tested.	Support collaborative approaches to the development and use of tested quality and performance measures, especially across federal and state programs.
Oversight and efforts to ensure accountability and improvements in the performance of Medicaid and CHIP.	Establish and monitor key Medicaid and CHIP performance standards, ensure programs meet minimum performance standards, and reward high performance.
There is a lack of consistent and coordinated leadership at federal and state levels for oral health programs.	Ensure that federal oral health leaders have education and experience qualifications consistent with professional standards applied to other health disciplines.

Source: Crall, J. J., & Vujicic, M. (2020). Children’s oral health: Progress, policy development, and priorities for continued improvement. *Health Affairs, 39*(10), 1762–1769. 10.1377/hlthaff.2020.00799. The information in this source is the basis of information presented in this table

At the national level, Healthy People 2030 focuses on reducing tooth decay and other oral health conditions and helping people get oral health care (Office of Disease Prevention and Health Promotion, 2022). Several federal programs have also developed outcome and performance measures for tracking progress. The Title V Maternal and Child Health (MCH) Services Block Grant Program includes one national outcome measure and two performance measures related to oral health (Lu et al., 2015). In 2010, CMS launched the Oral Health Initiative and documented national and state-specific improvements in use of preventive oral health care by children enrolled in Medicaid (Centers for Medicare & Medicaid Services, 2020). The Office of Head Start has also established Program Performance Standards to ensure enrolled children’s teeth are brushed with fluoride toothpaste daily and that they have a dental home (Administration for Children & Families, 2023). Other MCH programs, such as the Maternal, Infant, and Early Childhood Home Visiting Program, Special Supplemental Nutrition Program for Women, Infants, and Children (WIC), and Healthy Start, should also integrate oral health into their programs.

In addition to the focus on dental services in traditional settings, the Community Preventive Services Task Force recommends CWF and school-based programs to deliver dental sealants based on strong evidence of their effectiveness in reducing tooth decay (Community Preventive Services Task Force, 2017). The U.S. Preventive Services Task Force also recommends that primary care clinicians prescribe oral fluoride supplements for children aged 6 months to 5 years whose water supply is deficient in fluoride (Davidson et al., 2021).

*Oral Health in America: Advances and Challenges* identified three broad-ranging concepts influencing oral health and disease outcomes (National Institutes of Health, 2021). First, oral health must be regarded as integral to overall health and should be embedded in the broad framework of whole-body health. Second, community conditions, poverty, and limited health literacy make it difficult for families to engage in healthy habits, use community-level and individual preventive interventions, and access treatment promptly. Accordingly, the social and commercial determinants of health need to be addressed. And finally, the separation of dentistry from overall health care has led to systems of oral health care financing and delivery that limit access to care and perpetuate disparities in oral health. Addressing these broad challenges will require leadership, advocacy, and ongoing efforts to develop policies, programs, and systems, and these should remain priorities for achieving future oral health improvements for the entire population. More than 30 years have elapsed since the landmark Public Health Service Workshop on the oral health of mothers and children. Therefore, it is time for the MCHB to take steps to convene a similar workshop to develop a framework for future actions.
