# Correction to: Oral Health of Women and Children: Progress, Challenges, and Priorities

**DOI:** 10.1007/s10995-023-03766-6

**Published:** 2023-09-02

**Authors:** Jayanth Kumar, James J. Crall, Katrina Holt

**Affiliations:** 1 Department of Public Health, MS 7208, 1616 Capitol Avenue, Sacramento, CA, 95814 USA; 2 UCLA School of Dentistry, Los Angeles, CA, 90095 USA; 3 Department of Pediatrics, National Maternal and Child Oral Health Resource Center, Georgetown University, 37th and O Streets, N.W, Washington, DC, 20057 USA

Correction to: Maternal and Child Health Journal

10.1007/s10995-023-03757-7

The article ‘‘Oral Health of Women and Children: Progress, Challenges, and Priorities’’, written by Jayanth Kumar, James J. Crall and Katrina Holt was originally published electronically on the publisher’s internet portal on 21 July 2023 without open access. With the author(s) decision to opt for Open Choice, the copyright of the article changed on 17 August 2023 to The Author(s) 2023 and the article is forthwith distributed under a Creative Commons Attribution 4.0 International License, which permits use, sharing, adaptation, distribution and reproduction in any medium or format, as long as you give appropriate credit to the original author(s) and the source, provide a link to the Creative Commons licence and indicate if changes were made. The images or other third-party materials in this article are included in the articles Creative Commons licence, unless indicated otherwise in a credit line to the material. If material is not included in the articles Creative Commons licence and your intended use is not permitted by statutory regulation or exceeds the permitted use, you will need to obtain permission directly from the copyright holder. To view a copy of this licence, visit http://creativecommons.org/licenses/by/4.0.

